# Acomys cahirinus develop lung stroma distortion but not fibrosis after bleomycin-induced injury

**DOI:** 10.1186/s41232-025-00391-4

**Published:** 2025-08-29

**Authors:** Nataliya A. Basalova, Vladimir S. Popov, Yulia G. Antropova, Natalia V. Danilova, Victoria N. Biryukova, Uliana D. Dyachkova, Maksim A. Vigovskiy, Olga A. Grigorieva, Natalia I. Kalinina, Anastasia Yu. Efimenko

**Affiliations:** 1 Centre for Regenerative Medicine, Medical Research and Education Institute, Lomonosov Moscow State University, Moscow, 119234 Russia; 2 Faculty of Medicine, Medical Research and Education Institute, Lomonosov Moscow State University, Moscow, 119234 Russia

**Keywords:** Pulmonary fibrosis, Acomys cahirinus, Myofibroblasts, Extracellular matrix (ECM), Bleomycin hydrolase

## Abstract

**Background:**

Spiny mice (*Acomys sp*.) have a unique ability of scarless regeneration. Therefore, the transfer of models used in convenient laboratory mice to study fibrosis could be a prospective approach, enabling the identification of novel antifibrotic therapies.

**Methods:**

In this study, we first applied a model of bleomycin-induced pulmonary fibrosis in *Acomys cahirinus (Acomys)*, using *Mus musculus* C57BL/6 (*Mus*) as a control. Changes in lung tissue density were assessed using magnetic resonance imaging (MRI). The severity of fibrosis in lung tissue, as well as the deposition of extracellular matrix components, was assessed by histochemical analysis and morphometry (hematoxylin and eosin, Van Gieson). Data on the content of the main profibrotic proteins of the extracellular matrix, including collagen types I and IV, fibronectin, and fibronectin with EDA domain, were additionally validated by dot blotting. Changes in the number and localization of the main cell types contributing to the development of fibrosis (myofibroblasts, activated stromal cells, epithelium, M2 macrophages, leukocytes) were assessed by immunohistochemical analysis and morphometry. Statistical analysis was performed using GraphPad Prism software. Kruskal–Wallis *H*-test with the Dunn test and Mann–Whitney test was used for comparison between groups. Differences were considered significant when *p < 0.05.

**Results:**

Our data demonstrate that *Acomys* can survive high doses of bleomycin, which are sub-lethal and lethal for C57/Bl6 mice strain. In the head-to-head study, we performed an MRI to reveal changes in lung density as well as analyzed the morphology of *Mus* and *Acomys* lungs together with the identification of cell types required for fibrotic development. In contrast to *Mus*, *Acomys* demonstrated a decrease in respiratory regions upon bleomycin administration, but “classical” signs of fibrosis, such as fibrotic focuses or extracellular matrix accumulation, are detected only in small areas.

**Conclusions:**

The model of bleomycin-induced pulmonary fibrosis in *Acomys* is valid for the further investigation of possible mechanisms of resistance to damage-induced profibrotic stimuli.

**Supplementary Information:**

The online version contains supplementary material available at 10.1186/s41232-025-00391-4.

## Background

The damage of organs and tissues in adult mammals should be followed by regeneration, but often fails and deregulates, resulting in progressive loss of tissue function due to the development of fibrosis, a process characterized by an excessive extracellular matrix (ECM) deposition and remodeling to the dense scar areas. Chronic fibrosis can affect tissues throughout the body, ultimately leading to organ failure and death. In the lungs, the replacement of the airy alveolar tissue by a stiff fibrotic matrix severely impairs respiratory function and, therefore, compromises the overall quality of life of the patient. Regardless of the trigger, which has initiated the fibrotic lung remodeling, the end-stage pulmonary fibrosis persists in the list of unmet medical needs. Several approaches of regenerative medicine have emerged to restore the damaged pulmonary tissue and recover its functions; however, there is a significant demand for the relevant animal models of lung regeneration after damage.

Rodents within the genus *Acomys* (e.g., egyptian spiny mouse, *Acomys cahirinus*) were first reported to have the unique ability to scar-free skin regeneration after injury interpreted as skin autotomy in mammals [1]. Early insights into *Acomys* regeneration have come from dermal wound healing studies that include full-thickness biopsy punches of the skin and ear and thermal burns of the skin [1–5]. However, recently the scarless regeneration of the complex tissues in *Acomys* was demonstrated for a wide range of organs including heart [6], musculoskeletal system [7], spinal cord [8] and kidneys [9, 10] (reviewed in detail in [11]. Among possible cellular mechanisms involved in the facilitation of regeneration in *Acomys*, the contribution of macrophages is thoroughly discussed along with a debilitated inflammatory response [10–13]. Stromal cells isolated from *Acomys* tissues were also shown to exhibit unique antifibrotic behavior [14]. Tomasso et al. (2023) demonstrated the role of ERK-dependent signaling in the balance between fibrosis and regeneration using an ear punch model in *Acomys* [15]. Important to note that during the healing process in *Acomys*, many of the cell types involved in the fibrotic response are transiently activating, but they exhibit distinctive transcriptomic patterns and functional capabilities promoting tissue regeneration rather than scarring [16]. These intriguing findings make *Acomys* a promising model organism to unravel the evolutionary determined mechanisms preventing the fibrosis-driven solid organ failure.

Remarkably, there is a lack of data regarding pulmonary fibrosis in *Acomys*. Some differences in respiratory physiology between *Acomys* and *Mus* were demonstrated, but their contribution to the unique regenerative capacity of *Acomys* was uncertain [17]. In rodents, several models of pulmonary fibrosis were developed, including silicosis and asbestosis [18–20], lung radiation exposure model [21, 22] as well as transgenic and humanized models in *Mus* [23]. However, the most extensively used and well-studied model represents the toxic damage of lung epithelium by the intratracheal introduction of bleomycin followed by the inflammatory response, activation of lung stromal cells, and fibrotic foci formation [24–26]. This model reproduces the key aspects of lung fibrotic remodeling, including the attraction of immune cells as well as the injury-induced accumulation of myofibroblasts.

Since the modeling of fibrosis in *Acomys* could provide an opportunity for discovery of novel antifibrotic therapies, we have developed and validated a model of bleomycin-induced pulmonary fibrosis in *Acomys*. Our data demonstrate that these animals develop low signs of fibrosis only upon administration of extremely high doses of bleomycin lethal for *Mus* and demonstrate distinctive cellular responses to lung injury.

## Materials and methods

### Bleomycin-induced pulmonary fibrosis model in Mus and Acomys

The work was performed on male C57BL/6 mice, 9- to 20-week-old, weighing 25 ± 1 g, obtained from Laboratory Animal Breeding Centre “Puschino” (Russian Federation). Males and females *Acomys cahirinus* from 10- to 14 weeks old, weighing 37 ± 5 g were primarily obtained from Moscow Zoo and then bred in the laboratory animal facility of the Faculty of Medicine, Medical Research and Education Institute, Lomonosov MSU. Animal housing and research procedures were conducted in compliance with Directive 2010/63/EU and approved by the Bioethical Committee of MSU, approval number 3.5 from 17 May 2024.

For pulmonary fibrosis modeling, bleomycin (Bleocin “Nippon Kayaku Co., LTD”, Japan, #PN0113322/01) in sterile PBS (30 μl) was injected intratracheally once (transoral instillation). We used dose of 3 U/kg on *Mus* (*n* = 3), or doses of 3 U/kg (*n* = 4), 5 U/kg (*n* = 4), and 15 U/kg (*n* = 3) on *Acomys*. After 28 days, animals (bleomycin treated and intact (*n* = 3 both for *Mus* and *Acomys*)) were euthanized with a tenfold dose of anesthetic i.p. injection, and lung tissues were collected for analysis.

### Magnetic resonance imaging (MRI)

MRI was performed using the ClinScan 7 T device, Bruker Biospin, USA. All mice underwent MRI at 0, 7, 14, and 27 days from the start of the experiment. MRI studies were conducted under inhalation anesthesia (Aerrane, Baxter HealthCare Corporation, USA) supplied by the animal anesthesia system (E-Z-7000 Classic System, E-Z-Anesthesia® Systems, USA): 3.5–4% isoflurane mix with atmospheric air for the induction of anesthesia and 2–2.5% isoflurane/air mix for its maintenance. Vital activity was monitored using a respiratory cycle monitor. Lung imaging was performed in T2 weighted mode with the suppression of the signal from adipose tissue using the Turbo Spin Echo sequence with the following parameters: TR = 1175 ms, TE = 55 ms, Echo train length = 8, FOV 42 × 60 mm, base resolution 216 × 384. The imaging analysis was carried out using the ImageJ program. We used only images of slices that passed through the chest in the frontal plane (*n* = 3–10 for each animal).

### Histological and immunostaining studies

Samples of lung tissue fixed with 10% neutral formalin were embedded in paraffin. Paraffin blocks were cut on a microtome (section thickness 1 μm); sections were mounted on glasses coated with polylysine (Menzel-Glaser Polysine Slides, Thermo FS, USA). Sections were dewaxed, stained, and embedded in synthetic polymer (CS703, Dako, USA) or Aqua polymount for IHC and analyzed blindly using a Leica DM600Β microscope (Leica Microsystems GmbH, Germany) equipped with a Leica DFC 420C digital camera (Leica Microsystems GmbH, Germany). To analyze the general structure of lung tissue, sections were stained with hematoxylin and eosin (H&E, Panreac, USA). The degree of collagen formation was assessed by picrosirius red staining with subsequent differentiation in 0.01 N hydrochloric acid. To assess the severity of fibrotic changes, Van Gieson’s staining of sections (Artisan, Dako, USA) was used.

For IHC analysis, antigens on sections were unmasked in citrate or TE buffer at 95 °C for 20 min. To reduce the background fluorescence, the samples were treated with 50 mM ammonium acetate, then the nonspecific binding was blocked with the normal 10% serum of the animal donor of the second antibodies (Abcam, UK) in 1% BSA (PanEco, Russia). To detect the targets, first antibodies FAPα (Bioss, bs-5758R, USA), alpha-smooth muscle actin (αSMA, abcam, ab5694, UK or Biolegend, 904601, UK), CD206 (abcam, ab64693, UK), CD45 (abcam, ab10558, UK), bleomycin hydrolase (BLMH) (Cloud Clone, PAC220Mu01, US), PCNA (ThermoFisher, PA5-27214, USA), and E-cadherin (abcam, ab53033, UK) overnight. The detection of the first antibodies was carried out using second antibodies conjugated with a fluorescent label Alexa 488 (A11034, A11001, Invitrogen, USA), Alexa 594 (A21203, A11032, Invitrogen, USA), and Alexa Plus 647 (A32733, A32728, Invitrogen, USA). Nuclei were counterstained with DAPI solution (Sigma, USA). Microscopic examination was carried out on a Leica DMi8 microscope equipped with a Leica DFC 7000 T camera (Leica Microsystems GmbH, Germany) using representative fields of view for obtaining photographs. Image processing and analysis (*n* ≥ 5 for each sample in case of quantitative analysis) were performed using LasX software (Leica Microsystems GmbH, Germany) and FiJi ((Fiji is Just) ImageJ 2.0.0-rc-68/1.53t/Java 1.8.0_172 (64-bit)).

### Dot blotting (dot-ELISA)

Lung tissue was frozen in liquid nitrogen and then diminished in 2% SDS solution. The protein in the lysate was quantified using the micro-BCA protein assay (Thermo Fisher Scientific, USA). Samples were applied in triplicate at 1 μl to a nitrocellulose membrane (Amersham) and air-dried. HEK293 lysates were used as a negative control, hTERT MSC lysates (ATCC, ASC52telo) as a positive control. The subsequent immunodetection included non-specific blocking with 5% dry milk on PBS and overnight incubation of membranes with primary antibody against collagen I (ab34710, abcam, UK), collagen IV (PA 1–36,063, ThermoFisher, USA), fibronectin (ab2413, abcam, UK) or EDA-fibronectin (ab6328, abcam, UK). After PBST washes, the membranes were incubated with horseradish peroxidase (HRP)-labeled secondary antibodies (P-RAMIss, P‐GARIss, Imtek, Russia) for 2 h. The labeled proteins were visualized with a ChemiDocTM Touch imaging system (Bio-Rad Laboratories, USA) using an enhanced chemiluminescence kit (Pierce, Thermo Fisher Scientific, USA). To normalize the obtained values of the protein of interest in the case of dot blot, we used the values of the amount of protein obtained by staining the membrane with Amido black.

### Statistical analysis

Statistical analysis was performed using GraphPad Prism software. Experimental data were expressed as median (± interquartile range). Kruskal–Wallis *H*-test with the Dunn test was used for multiple comparisons. Mann–Whitney test was used for comparison between two groups. Differences were considered significant when *p* < 0.05; *p*-values are represented on figures.

## Results

### Acomys resist higher doses of bleomycin due to the high expression of bleomycin hydrolase

**Fig. 1:**
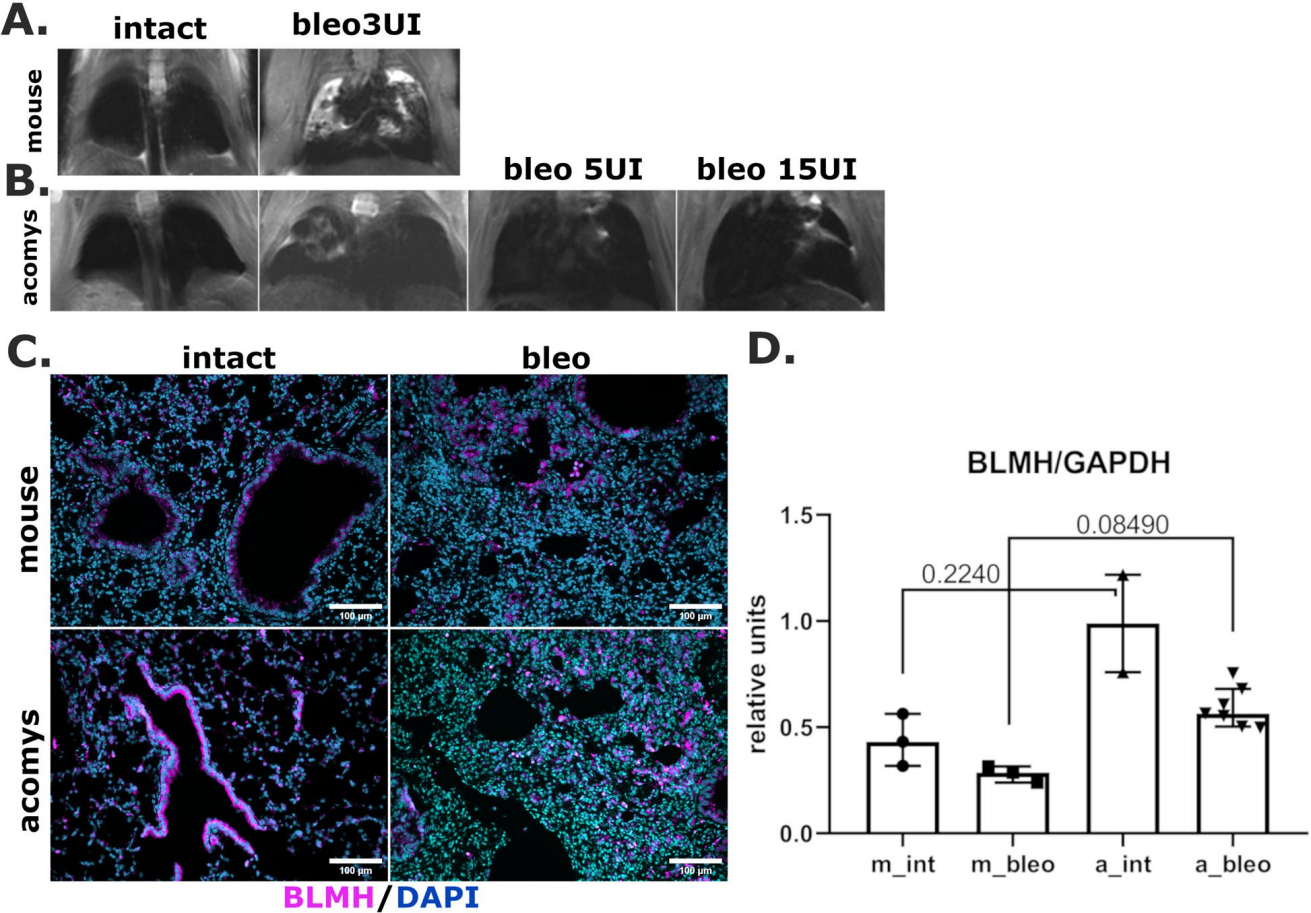
*Acomys* resist higher doses of bleomycin. **A**, **B** Representative images of MRI for lung tissue of *Mus* (**A**) and *Acomys* (**B**). **C** Representative images of pulmonary tissue immunocytochemical analysis for bleomycin hydrolase (BLMH); purple—BLMH, blue—DAPI. Scale bar = 100 μm. **D** Quantification of bleomycin hydrolase (BLMH), normalized to GAPDH, in pulmonary tissue, western blot. Data presented as median (± interquartile range). Kruskal-Wallis *H*-test with the Dunn test was used for multiple comparisons. Groups: m_int, a_int-lung tissue of intact *Mus* or *Acomys*; m_bleo, a_bleo-lung tissue of *Mus* or *Acomys* treated by bleomycin

Mouse lungs develop fibrosis after bleomycin-induced injury. Thus, the lung structure of *Mus* demonstrates prominent changes 28 days after intratracheal application of 3 U/kg of bleomycin. These changes are characterized by MRI signs of increased lung density (Fig. 1A). In contrast to *Mus*, the intratracheal application of bleomycin did not cause any significant changes in the lung density of *Acomys* as analyzed by MRI (Fig. 1B). Furthermore, *Acomys* have survived even higher doses of bleomycin (5 or 15 U/kg), which are sub-lethal or lethal for *Mus*, with only minor changes in the lung density on MRI (Fig. 1B).

To reveal if the ability of *Acomys* to survive the extreme bleomycin doses could be explained by the intrinsic high expression of the bleomycin hydrolase, we have compared the expression of this enzyme in both rodent species. Both *Mus* and *Acomys* expressed the bleomycin hydrolase in the epithelial cells of terminal bronchi as well as in selected cells of the respiratory regions of the lungs (Fig. 1C). Semi-quantitative analysis has revealed that *Acomys* lungs contained 2 times more bleomycin hydrolase compared to *Mus*. Bleomycin introduction caused a tendency to the decrease of this enzyme content in both species (Fig. 1D, Fig. S1), possibly due to the massive elimination of epithelial cells, as a source of bleomycin hydrolase caused by the application of this drug. These data indicate that the ability of *Acomys* to sustain the high bleomycin doses may correlate with the high expression of the bleomycin hydrolase.

### Acomys lungs fail to develop prominent fibrosis in response to bleomycin

**Fig. 2:**
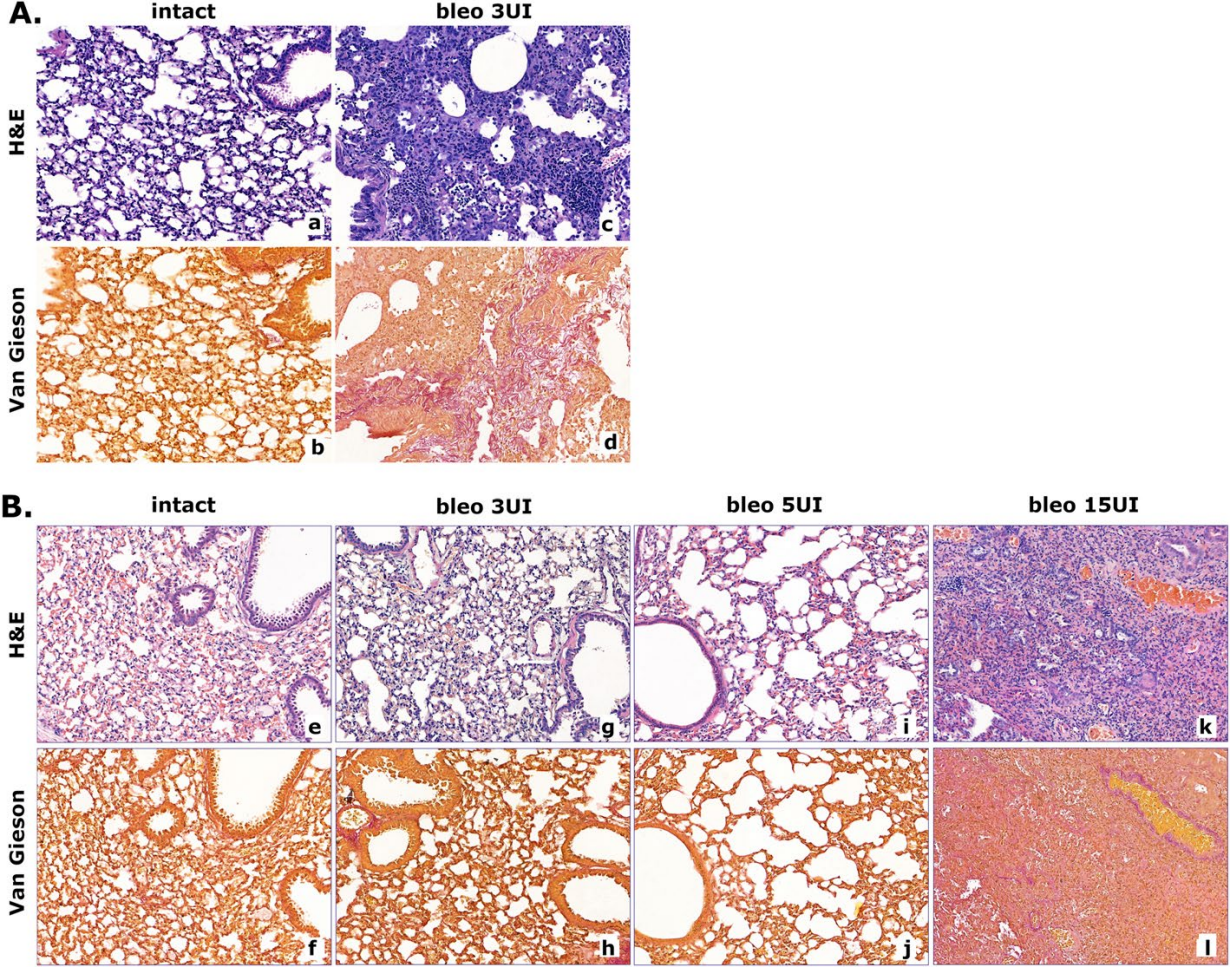
Histological changes of lung tissue of *Mus* (**A**) and *Acomys* (**B**) after bleomycin introduction. **a**, **b** Lung tissue of intact *Mus*: alveoli lumens are clear, the alveoli are not dilated, normal bronchial epithelium, no fibrosis on Van Gieson stain. **c**, **d** Lung tissue of *Mus* treated by 3 U/kg of bleomycin: atelectasis, bronchial epithelium hyperplasia, severe fibrosis positive with Van Gieson staining (reddish color). **e**, **f** Lung tissue of intact *Acomys*: alveoli lumens are smaller and collapsed in favor of capillary congestion, normal bronchial epithelium, no fibrosis on Van Gieson stain. **g**, **h** Lung tissue of *Acomys* after intratracheal application of 3 U/kg of bleomycin: acute capillary congestion, normal bronchial epithelium, no fibrosis on Van Gieson stain. **i**, **j** Lung tissue of *Acomys* treated by 5 U/kg of bleomycin: acute capillary congestion, slight enlargement of alveoli and flattening of the bronchial epithelium, no fibrosis on Van Gieson stain. **k**, **l** Lung tissue of *Acomys* treated by 15 U/kg of bleomycin: acute congestion, atelectasis, and thin fibrous septa, positive with Van Gieson staining. **a**, **c**, **e**, **g**, **i**, **k** Hematoxylin and eosin (H&E), × 20. **b**, **d**, **f**, **h**, **j**, **l** Van Gieson fibrous stain, × 20

Sponge-like respiratory regions of *Mus* intact lungs are composed of airy alveoli interspersed by the blood vessels of various diameters (Fig. 2A(a, b)). Lungs of *Acomys* demonstrate a peculiar morphology: the blood vessels, which surround alveoli, are notably wider compared to mouse tissue and are easily recognized by their content of red blood cells (Fig. 2B(e, f)). In response to the bleomycin-induced injury, *Mus* lungs develop morphological features specific to fibrosis, including thick alveolar walls and fibrotic focuses, which merge and form so-called “obliteration fields” (Fig. 2A(c, d)).

Bleomycin-induced injury caused a remarkable distortion of the lung structure of *Acomys* (Fig. 2B). The area occupied by respiratory competent tissue decreases 28 days after the treatment with bleomycin, in a dose-dependent mode. However, instead of “classical” fibrosis signs, we observed the prominent increase of blood vessel width within the respiratory regions of the lungs. Expanded blood vessels were accompanied by vast cellular infiltration. Observed changes were minimal at the 3 U/kg dose of bleomycin and most eminent after the introduction of 15 U/kg of the drug (Fig. 2B(k, l)).

**Fig. 3:**
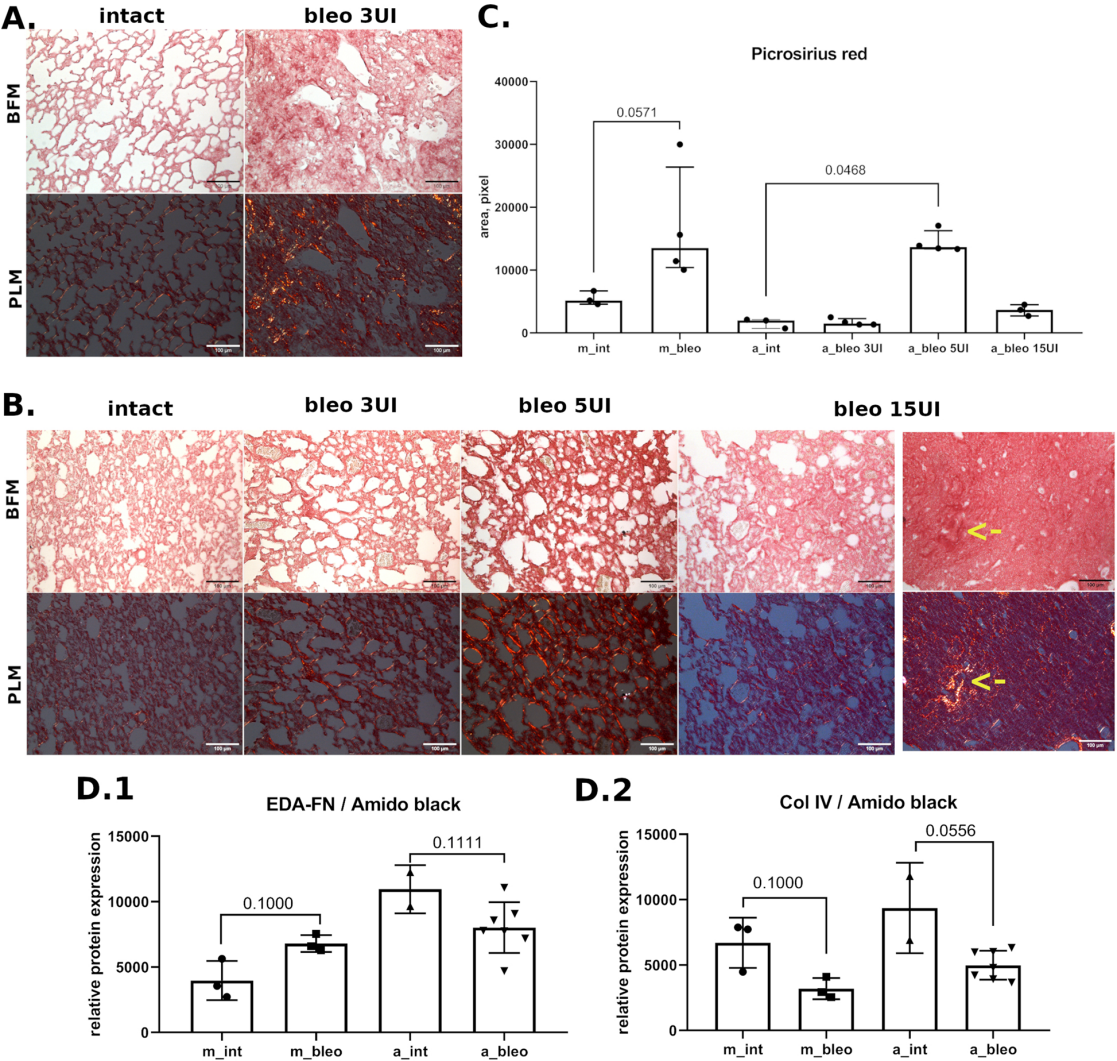
Accumulation of extracellular matrix proteins in the lung tissue of *Mus* and *Acomys* after bleomycin introduction. **A**, **B** Representative images for lung tissue of *Mus* (**A**) and *Acomys* (**B**); the arrows point to the location of zones morphologically similar to obliteration fields. Picrosirius red staining. BFM—bright field microscopy, PLM—polarized light microscopy. **C** Quantification of positive polarized areas in pulmonary tissue. Data present as median (± interquartile range). Mann-Whitney test was used for pairwise comparison. **D** Quantification of fibronectin with EDA domain (EDA-FN, D1) or collagen type IV (Col IV, D2) in pulmonary tissue, dot blot. Data presented as median (± interquartile range). Mann-Whitney test was used for pairwise comparison. Groups: m_int, a_int—lung tissue of intact *Mus* or *Acomys*; m_bleo, a_bleo—lung tissue of *Mus* or *Acomys* treated by bleomycin

Consistently with these observations, the bleomycin introduction caused a less prominent increase in the area occupied by collagen fibers in *Acomys* compared to *Mus* (Fig. 3). Thus, the area of deposited collagen in *Mus* lungs has increased about 2 times 28 days after the treatment by bleomycin (Fig. 3A, C). Under the same experimental conditions, the collagen accumulation has not increased in *Acomys* (Fig. 3B, C). Notably, higher bleomycin doses (5 and 15 U/kg) caused the elevation of collagen accumulation. Surprisingly, the most prominent collagen deposition was observed in animals treated with 5 U/kg of bleomycin, specifically within thickened interalveolar septa (Fig. 3B, C). In *Acomys*, which have received 15 U/kg of bleomycin, only a few zones morphologically similar to the obliteration fields were found (Fig. 3B, arrows). Using polarized microscopy, we revealed that deposited collagen is mostly represented by the mature fibers (Fig. 3B).

Observed fibrotic changes of lung respiratory regions in *Mus* were accompanied by changes in the ECM content, measured by western-blot. Despite the content of type I collagen not having changed (Fig. S2B), the marker protein of profibrotic matrix fibronectin, including its splice form EDA-fibronectin, has significantly increased (Fig. 3D.1, S2 A, S3 A). It is interesting to note that the amount of EDA-fibronectin in intact *Acomys* is three times higher than in intact mice (Fig. 3D.1, Fig. S3A). At the same time, the quantity of type IV collagen has decreased in both studied species (Fig. 3D.2, Fig. S2B). In contrast, the lung tissue distortion in *Acomys* was not associated with the increase of any tested ECM protein. Furthermore, we observed a tendency to decline in the content of those proteins (Fig. 3D, Figs. S2 and S3).

Taken together, these data suggest that *Acomys* fail to develop pulmonary fibrosis in response to the same dose of bleomycin as *Mus*.

### Acomys do not demonstrate myofibroblast accumulation in damaged lungs

**Fig. 4:**
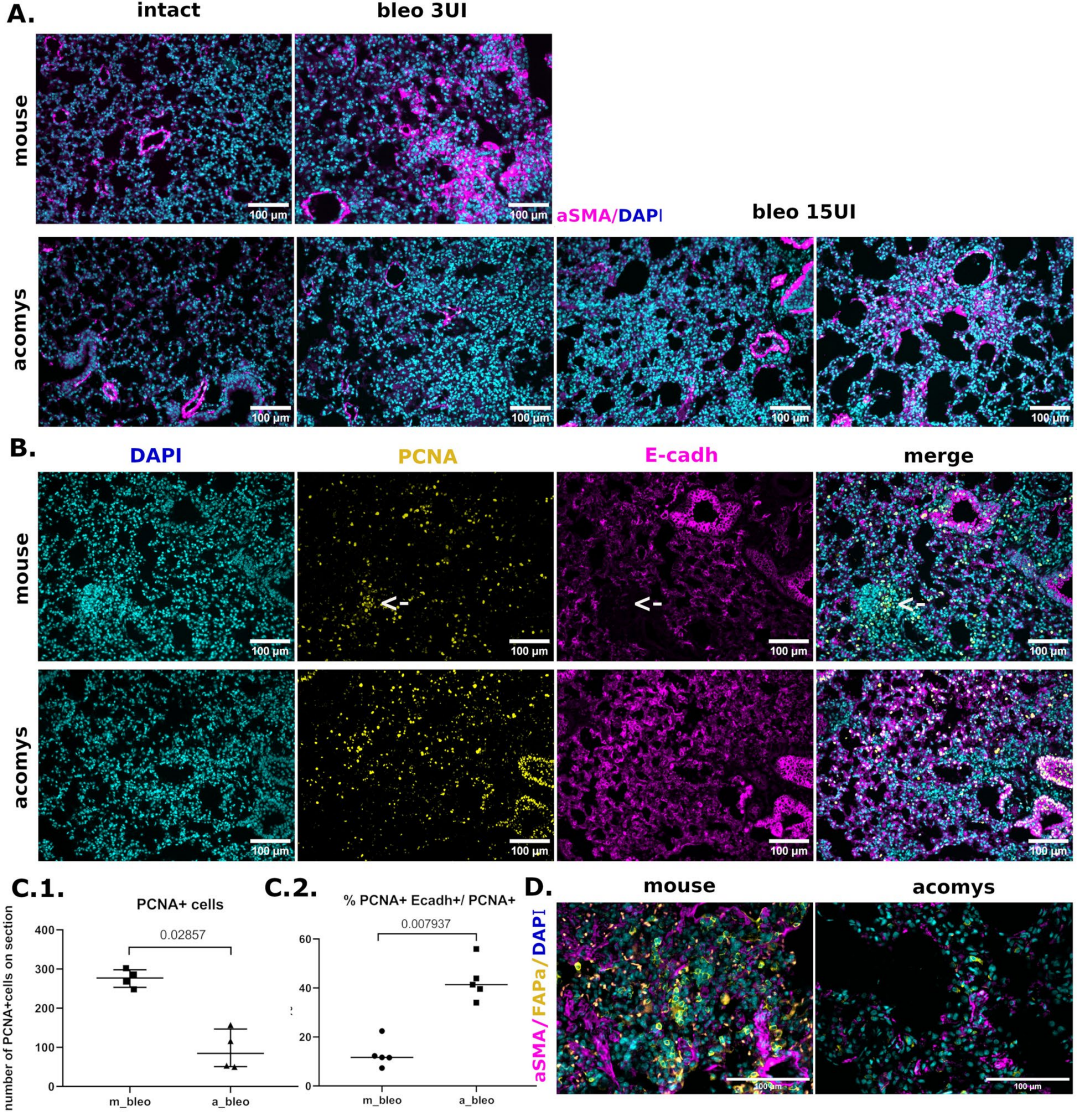
*Acomys* do not exhibit an increase in activated fibroblasts and myofibroblasts. **A** Representative images for lung tissue of *Mus* (top) and *Acomys* (bottom), IHC, blue—DAPI, purple—Asma. **B** Representative images for lung tissue of *Mus* and *Acomys*, IHC; blue—DAPI, purple—E-cadherin, yellow—PCNA. **C** Quantification of total amount of PCNA + (**C1**) or PCNA + E-cadherin + population (**C2**) in pulmonary tissue. Data present as median (± interquartile range). Mann-Whitney test was used for pairwise comparison. Groups: m-bleo, a-bleo—lung tissue of *Mus* or *Acomys* treated by bleomycin. **D** Representative images for lung tissue of *Mus* and *Acomys*, IHC; C: blue—DAPI, purple—aSMA, yellow—FAPα

Since the fibrosis development depends on the recruitment of specific driver cell populations, including myofibroblasts and activated stromal cells, we have compared their content in the lungs of *Mus* and *Acomys* 28 days after bleomycin-induced injury. As expected, in *Mus* lung the number of αSMA expressing myofibroblasts has increased severely (Fig. 4A). Furthermore, the injury has caused the increase in the number of proliferating E-cadherin negative cells (Fig. 4B(C1, C2)). These non-epithelial, presumably stromal cells were distributed dispersedly as well as within specific compact groups, which correlated well with the formation of fibrotic foci by activated stromal cells and myofibroblasts (Fig. 4B, arrows) [26].

In contrast, the bleomycin-induced injury did not cause the accumulation of myofibroblasts in *Acomys* (Fig. 4A). Of note, the highest bleomycin dose led to the appearance of the rare zones enriched by myofibroblasts, likely associated with peribroncheal fibrosis (Fig. 4A, bleo 15UI right). Consistent with this observation, *Acomys* lungs contained fewer cells expressing the marker protein of activated fibroblasts (FAPα-positive cells) (Fig. 4D). At the same time, the proliferating cell marker, PCNA, was predominantly expressed by E-cadherin positive epithelial cells (Fig. 4B (C1, C2)). This indicated the important investment of epithelial proliferation in the increased cellularity of alveolar septa.

**Fig. 5:**
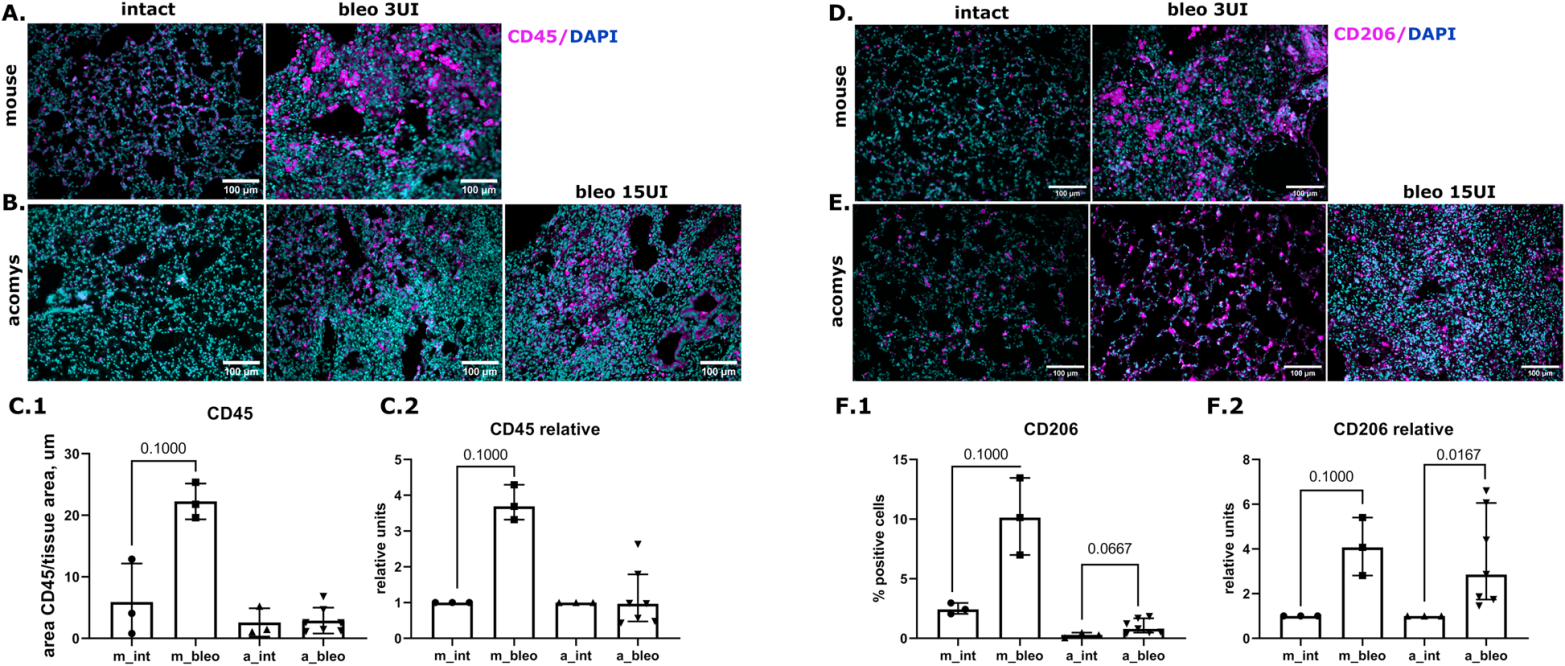
Macrophage infiltration of the lung tissue of *Mus* and *Acomys* after bleomycin introduction. **A**, **B** Representative images of CD45 expression in lung tissue of *Mus* (**A**) and *Acomys* (**B**), IHC, blue—DAPI, purple—CD45. **C** Quantification of CD45 + total area (**C1**) or normalized to the corresponding groups of intact animals (**C2**) in pulmonary tissue. Data present as median (± interquartile range). **D**, **E** Representative images of CD206 expression in lung tissue of *Mus* (**D**) and *Acomys* (**E**), IHC, blue—DAPI, purple—CD206. **F** Quantification of CD206 + cells in total or normalized to the corresponding groups of intact animals. Data presented as median (± interquartile range). Mann-Whitney test was used for pairwise comparison. Groups: m_int, a_int—lung tissue of intact *Mus* or *Acomys*; m_bleo, a_bleo—lung tissue of Mus or *Acomys* treated by bleomycin

Since specific subpopulations of immune cells were suggested to contribute to the ability of *Acomys* to resist fibrosis, we also have compared their accumulation in injured lungs. Thus, the area occupied by CD45 + leukocytes as well as by CD206 + macrophages in *Mus* lungs has increased 4 times in response to bleomycin-induced injury (Fig. 5, Fig. S4). Similarly, the increased number of CD206 + cells was also observed in *Acomys* lungs (Fig. 5E, F). However, the number of CD45 + leukocytes in *Acomys* lungs has not changed in response to the injury (Fig. 5B, C).

Together, these data indicate that bleomycin-induced injury caused distinctive cellular responses in examined rodent species.

## Discussion

This study reveals the possibility of inducing the development of lung tissue injury in *Acomys* (spiny mice). In contrast to conventional murine models, those that develop robust lung fibrosis following bleomycin-induced injury, *Acomys* exhibit damage with only minor signs of fibrosis even at the extreme drug doses, which are lethal for *Mus* C57Bl/6 strain. Observed changes on MRI were localized in the upper lobes of the lungs, which is typical for the bleomycin model [27]. Although breathing capacities were not specifically analyzed, *Acomys* did not demonstrate abnormal behavior, weight loss, decreased appetite, or other signs of dysadaptation.

Decreased bleomycin toxicity could be attributed to the elevated expression of bleomycin hydrolase by *Acomys*. However, this fact hardly explains their resistance to fibrosis: BALB/c mice demonstrate even higher relative levels of bleomycin hydrolase activity; however, they develop prominent pulmonary fibrosis in response to the bleomycin [28]. Although collagen deposition in BALB/c strain is two times less than in C57Bl/6 after the same dose of bleomycin [29], which is consistent with diminished collagen accumulation in *Acomys, the* obvious fibrotic response to bleomycin in both murine strains was clearly demonstrated.

Our data are consistent with previously published data, which indicate that fibrotic response is markedly reduced in *Acomys* [16]. There are several potential mechanisms responsible for diminished fibrosis, including species-specific ECM production and remodeling without collagen accumulation; a distorted immune response, favoring antifibrotic M2 macrophages; an antifibrotic cytokine environment, and rapid elimination of myofibroblasts as main fibrosis drivers.

Impaired collagen accumulation in *Acomys* lungs is accompanied by decreased accumulation of αSMA-positive myofibroblasts, consistent with the data obtained in the ear puncture model, which also demonstrates swift myofibroblast elimination. This could be explained by the rapid inactivation of Yap/Taz signaling, which prevents a persistence of the myofibroblast phenotype and their de-differentiation [30, 31]. Previously, we have shown that extracellular vesicles promote lung fibrosis resolution by accelerating myofibroblast de-differentiation [32]. Our current data also indicate that the diminished number of myofibroblasts is accompanied by a low quantity of FAPα + myofibroblast precursors. Notably, the collagen deposition in *Acomys* was found specifically in association with large blood vessels, suggesting that the vascular wall could serve as a source of collagen-producing cells, and these are differently regulated compared to other stromal cells.

Contrary to our expectation, we did not find a significant difference in CD206 + macrophage accumulation between *Acomys* and *Mus*. These cells were suggested for successful epimorphic regeneration without fibrosis [31]. Moreover, recent data show that CD206 can label tissue-resident macrophages that exhibit a proregenerative phenotype [12, 33]. However, the input of these cell types at the earlier time points and in vitro experiments on isolated subtypes of macrophages must be assessed before the conclusion about their involvement in the lung fibrosis development in *Acomys*.

We showed that *Acomys* demonstrate a tendency to decrease the production of ECM proteins, including type IV collagen and EDA-fibronectin upon bleomycin introduction. These data well correlate with the observation [14, 30] that *Acomys* dermal fibroblasts have a reduced ability to synthesize collagen type I, III, and IV. In addition, *Acomys* can readily restore normal collagen folding at the damage sites, in contrast to *Mus*, which form scars characterized by parallel collagen bundles [30, 34].

Interestingly, *Acomys* demonstrate higher basal expression of EDA-fibronectin in the lung tissue compared to *Mus*. Considering that this fibronectin isoform could be involved in the angiogenesis promotion [35], high EDA-fibronectin content well correlates with a high density of blood capillaries in *Acomys* lung. The increased intrinsic ability for angiogenesis in *Acomys* [3] could be responsible for a lung stroma distortion, resembling atelectasis seen in human lungs.

Indeed, at the highest bleomycin doses, *Acomys* develop remarkable lung stroma distortion rather than fibrosis. Taking into account that cells in *Acomys* are more predisposed to proliferation, we hypothesized that enormous cellularity in *Acomys* lungs could arise from the rapid division of stromal cells. On the contrary, the number of proliferating cells in *Acomys* appeared significantly less than in *Mus*. Furthermore, the overwhelming majority of PCNA + cells in *Acomys* lung express E-cadherin; therefore, representing epithelial cells. Active proliferation of the epithelium is required for rapid restoration of alveolocytes after damage and may depend on the Erk signaling pathway [15].

We would like to emphasize the key limitations of the study. In particular, due to the novelty of *Acomys* as a model object and slow reproductive rate of this species, our study had a small sample of animals. In this regard, it is necessary to conduct a study on an increased sample size to clarify the statistical difference in the data. In addition, the dynamics of the response of pulmonary tissue to the damage should be investigated for a more comprehensive understanding of the mechanisms underlying fibrosis development and resolution.

## Conclusion

To our knowledge, this study is the first to demonstrate that *Acomys* fail to develop well-established pulmonary fibrosis in the model of bleomycin-induced lung injury, even if high doses of bleomycin, sublethal and lethal for C57/Bl6 mice strain, are used. However, *Acomys* exhibit significant pathomorphological changes in lungs upon bleomycin administration, which can be described as a damage with only minor signs of fibrosis. These data suggest that this model is feasible for studying pulmonary damage in *Acomys*. Comparing directly the response of lung tissue to bleomycin-induced injury in *Mus* and *Acomys* we revealed the important differences between these species, including the lack of thickening alveolar walls and fibrotic foci formations in *Acomys*, together with less accumulation of myofibroblasts and collagens in lung stroma. These cellular mechanisms could be potentially responsible for the observed effects, but need to be further investigated. Our findings highlight *Acomys* as an interesting and promising animal model for exploring the pathogenesis of fibrosis initiation and progression, as well as possible mechanisms of resistance to damage-induced profibrotic stimuli for developing the novel antifibrotic therapeutic approaches.

## Supplementary Information

Supplementary Material 1: Figure S1. *Acomys cahirinus *resist higher doses of bleomycin. Figure S2. Accumulation of extracellular matrix proteins in the lung tissue of *Mus *or *Acomys* after bleomycin introduction. Figure S3. Accumulation of extracellular matrix proteins in the lung tissue *Mus *or *Acomys *after bleomycin introduction. Figure S4. Macrophage infiltration of the lung tissue of *Mus* or *Acomys* after bleomycin introduction.

## Data Availability

All data generated or analyzed during this study are included in this published article [and its supplementary information files].

## Abbreviations

αSMA: Alpha smooth muscle actin
BLMH: Bleomycin hydrolase
CD: Cluster of differentiation
ECM: Extracellular matrix
ERK: Extracellular signal-related kinase
FAPα: Fibroblast activation protein alpha
MRI: Magnetic resonance imaging
PCNA: Proliferating cell nuclear antigen
