# Supplementary material for: Acomys cahirinus develop lung stroma distortion but not fibrosis after bleomycin-induced injury
Source: Inflamm Regen. 2025 Aug 29;45:27. doi: 10.1186/s41232-025-00391-4 (PMC12395645; doi:10.1186/s41232-025-00391-4)
Supplement: Supplementary file 1 — Supplementary Material 1: Figure S1. Acomys cahirinus resist higher doses of bleomycin. Figure S2. Accumulation of extracellular matrix proteins in the lung tissue of Mus or Acomys after bleomycin introduction. Figure S3. Accumulation of extracellular matrix proteins in the lung tissue Mus or Acomys after bleomycin introduction. Figure S4. Macrophage infiltration of the lung tissue of Mus or Acomys after bleomycin introduction. [file 41232_2025_391_MOESM1_ESM.pdf]

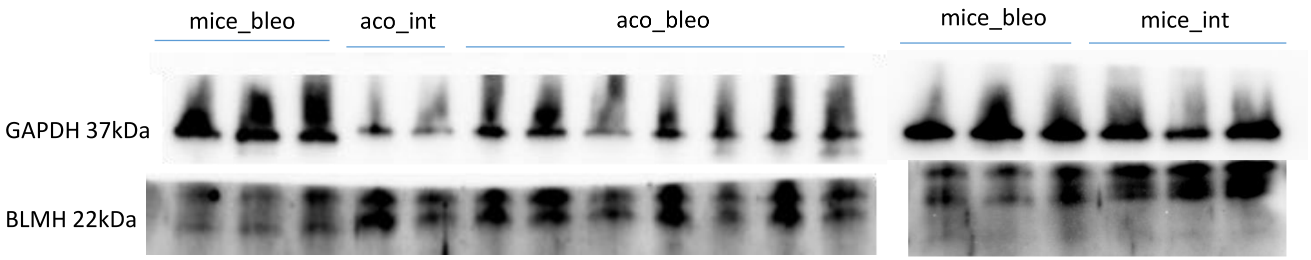

Figure S1 Acomys cahirinus resist higher doses of bleomycin
Representative images of the membrane with antibody against GAPDH (top) or bleomycin hydrolase (bottom).
Groups: mice_int, aco_int - lung tissue of intact *Mus* or *Acomys*; mice_bleo, aco_bleo - lung tissue of *Mus* or *Acomys* treated by bleomycin

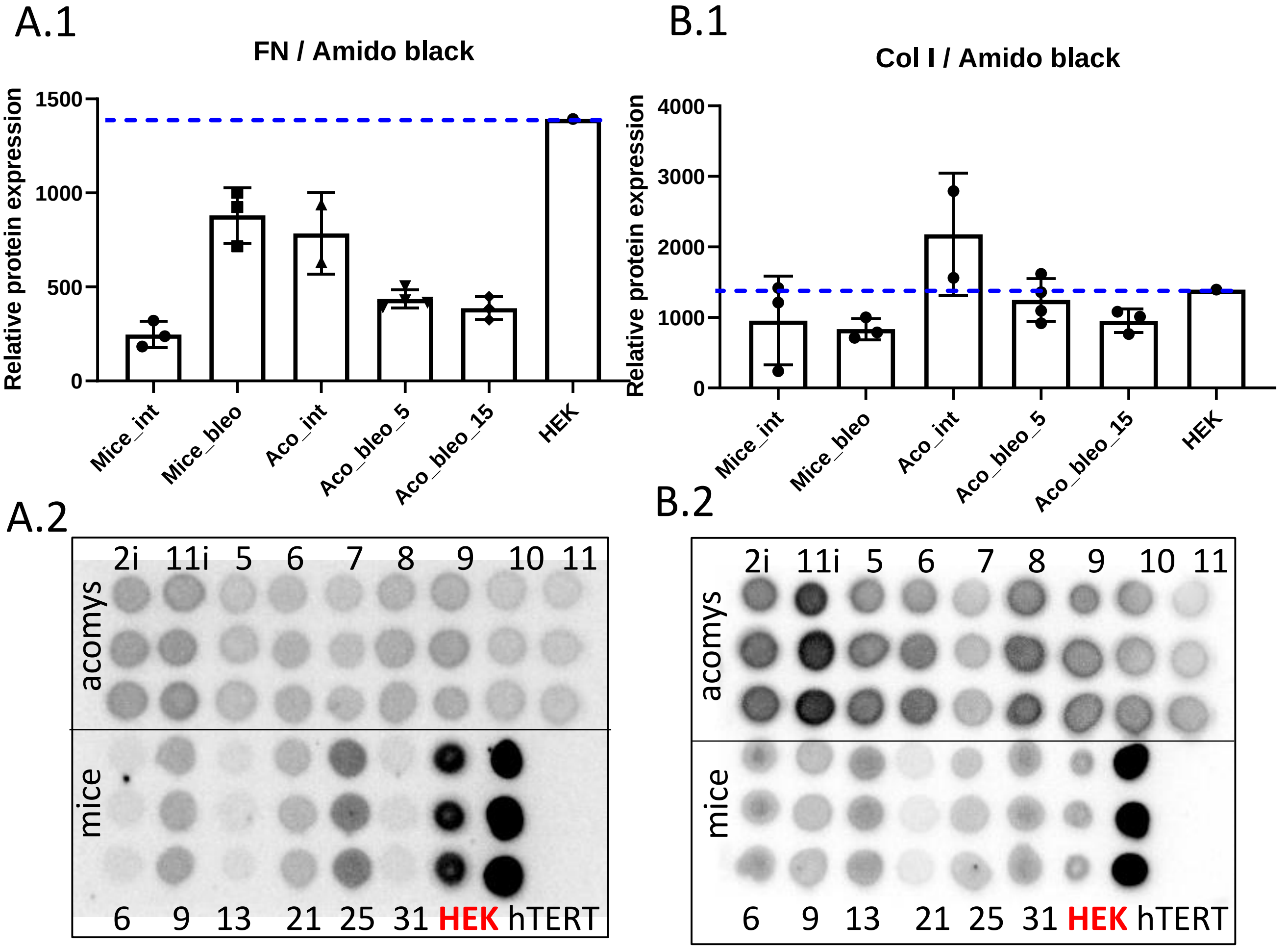

Figure S2 Accumulation of extracellular matrix proteins in the lung tissue of *Mus* or *Acomys* after bleomycin introduction.

A1, B1 - Quantification of fibronectin (FN, A1) or collagen type I (Col I, B1) in pulmonary tissue, dot blot. Data present as median (± interquartile range). Groups: mice_int, aco_int - lung tissue of intact *Mus* or *Acomys*; mice_bleo, aco_bleo - lung tissue of *Mus* or *Acomys* treated by bleomycin
A2, B2 - Representative images of the membrane with antibody against fibronectin (A2) or collagen type I (B2)

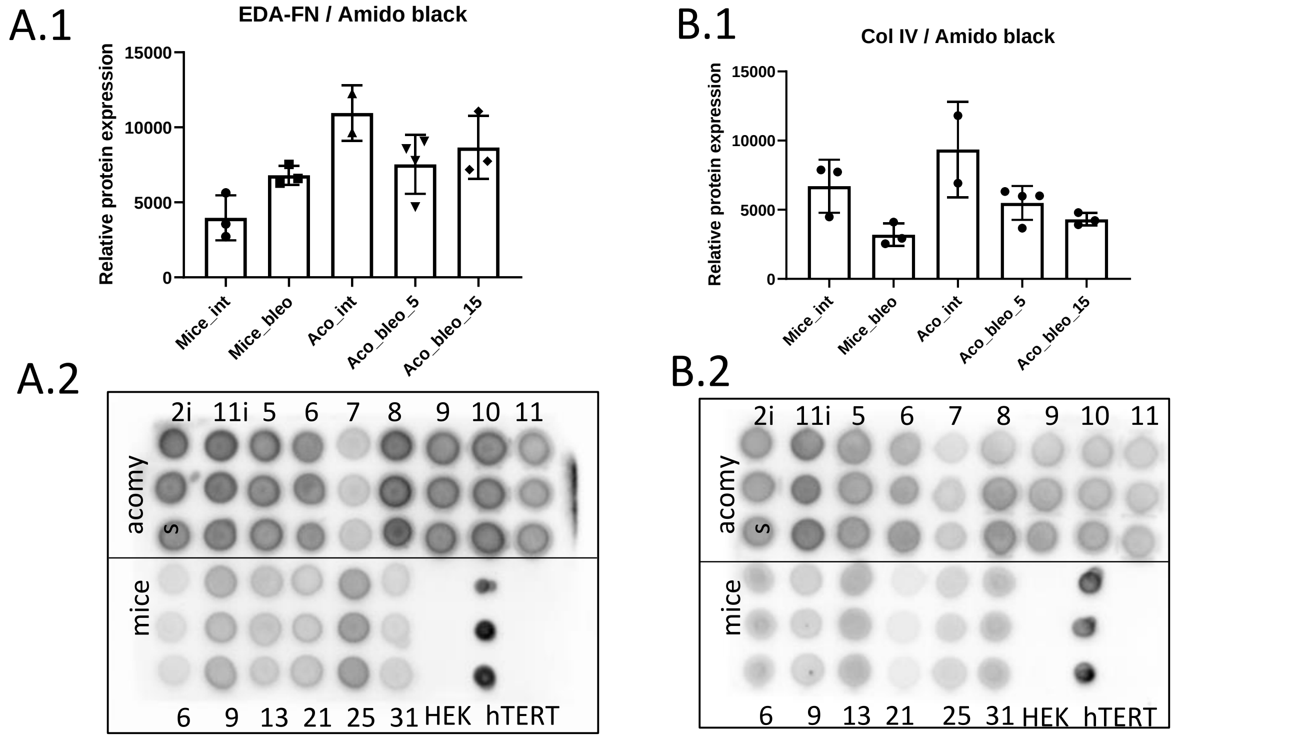

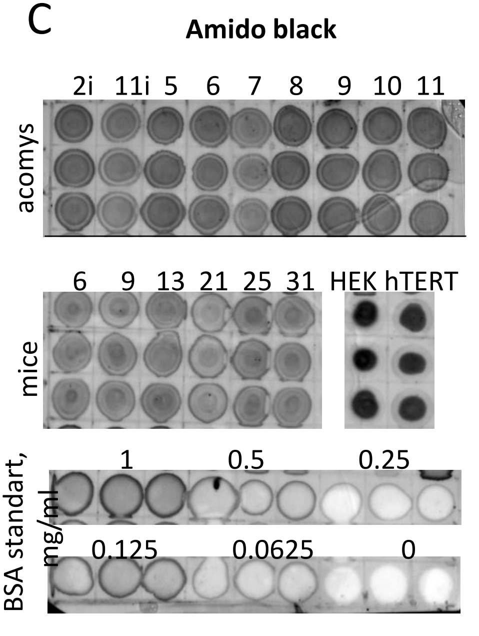

Figure S3 Accumulation of extracellular matrix proteins in the lung tissue *Mus* or *Acomys* after bleomycin introduction.

A1, B1 - Quantification of fibronectin with EDA domen (EDA-FN, A1) or collagen type IV (Col IV, B1) in pulmonary tissue, dot blot. Data present as median (± interquartile range).

Groups: mice_int, aco_int - lung tissue of intact *Mus* or *Acomys*; mice_bleo, aco_bleo - lung tissue of *Mus* or *Acomys* treated by bleomycin

A2, B2 - Representative images of the membrane with antibody against EDA-fibronectin (A2) or collagen type IV (B2)

C - Representative images of the membrane with Amido black staining

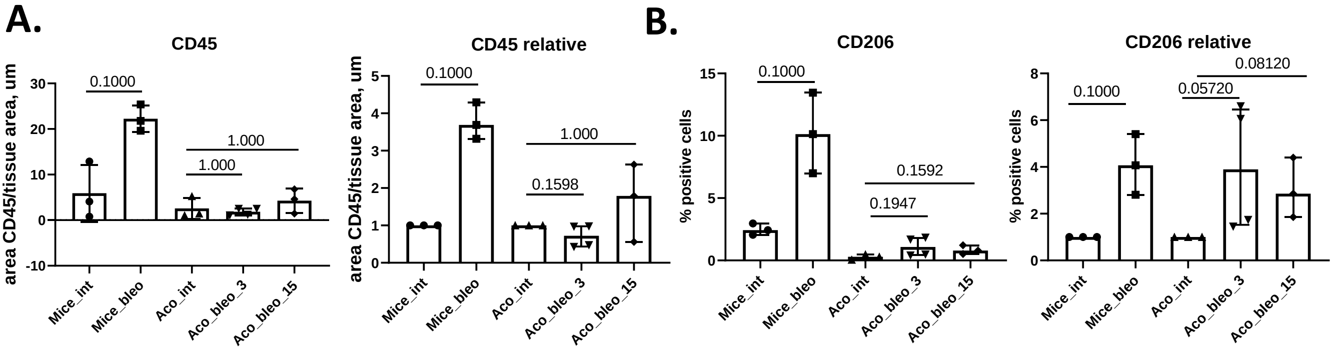

Figure S4. Macrophage infiltration of the lung tissue of *Mus* or *Acomys* after bleomycin introduction.

A - Quantification of CD45+ total area (left) or normalized to the corresponding groups of intact animals (right) in pulmonary tissue. Data present as median (± interquartile range).
B - Quantification of CD206+ cells in total (left) or normalized to the corresponding groups of intact animals (right). Data present as median (± interquartile range).
Groups: mice_int, aco_int - lung tissue of intact *Mus* or *Acomys*; mice_bleo, aco_bleo - lung tissue of *Mus* or *Acomys* treated by bleomycin
